# Fitness seascapes are necessary for realistic modeling of the evolutionary response to drug therapy

**DOI:** 10.1126/sciadv.adv1268

**Published:** 2025-06-11

**Authors:** Eshan S. King, Anna E. Stacy, Davis T. Weaver, Jeff Maltas, Rowan Barker-Clarke, Emily Dolson, Jacob G. Scott

**Affiliations:** ^1^ Case Western Reserve University School of Medicine, Cleveland, OH 44106, USA.; ^2^ Medical Scientist Training Program, Case Western Reserve University, Cleveland, OH 44106, USA; ^3^ Department of Physics, Case Western Reserve University, Cleveland, OH 44106, USA.; ^4^ Department of Translational Hematology-Oncology Research, Cleveland Clinic, Cleveland, OH 44106, USA.; ^5^ Department of Computer Science and Engineering, Michigan State University College of Engineering, East Lansing, MI 48824, USA.; ^6^ Ecology, Evolution, and Behavior Program, Michigan State University, East Lansing, MI 48824, USA.; ^7^ Department of Radiation Oncology, Cleveland Clinic, Cleveland, OH 44106, USA.

## Abstract

Pharmacokinetic (PK) and pharmacodynamic (PD) modeling of host-pathogen interactions has enhanced our understanding of drug resistance. However, how combinations of drug resistance mutations affect dose-response curves remains underappreciated in PK-PD studies. The fitness seascape model addresses this by extending the fitness landscape model to map genotypes to dose-response functions, enabling the study of evolution under fluctuating drug concentrations. Here, we present an empirical fitness seascape in *E. coli* harboring all combinations of four drug resistance mutations. Incorporating these data into PK-PD simulations of antibiotic treatment, we find that higher mutation supply increases the probability of resistance, and early adherence to the drug regimen is critical. In vitro studies further support the finding that the second dose in a drug regimen is important for preventing resistance. This work bridges empirical fitness seascapes and computational PK-PD studies, revealing insights into drug resistance.

## Teaser

Computational and experimental studies of drug resistance evolution give insights into why some bacteria become resistant.

## INTRODUCTION

Antimicrobial resistance is a persistent challenge that contributes to substantial global mortality and economic burden, with disproportionate impacts on those in low-resource settings (*1*). Understanding and predicting the emergence of drug resistance within and among human hosts is an intuitive path toward developing effective treatment strategies. The emergence of resistance is studied through an evolutionary lens, whereby novel genotypes of a disease agent emerge through a random process and are selected for due to their increased fitness. Recently, computational studies of the evolution of resistance have been used to optimize dosing schedules in silico, in vitro, and in clinical trials (*2*–*9*).

To understand, simulate, and predict evolution, researchers often use the fitness landscape model, which maps genotype to fitness (*10*–*16*). An assumption of the fitness landscape model is that, for a given landscape, the selection pressure is constant (*12*). Hence, canonical fitness landscapes cannot model evolution under continuously varying selection pressure, such as the rising and falling concentration of drug in a patient over time. This limitation of singular fitness landscapes precludes the modeling of evolutionary trade-offs, or fitness costs, where a mutant “trades” a lower growth rate in the absence of a drug in exchange for a higher growth rate at higher concentrations of the drug (*17*–*19*).

**Fig. 1.**
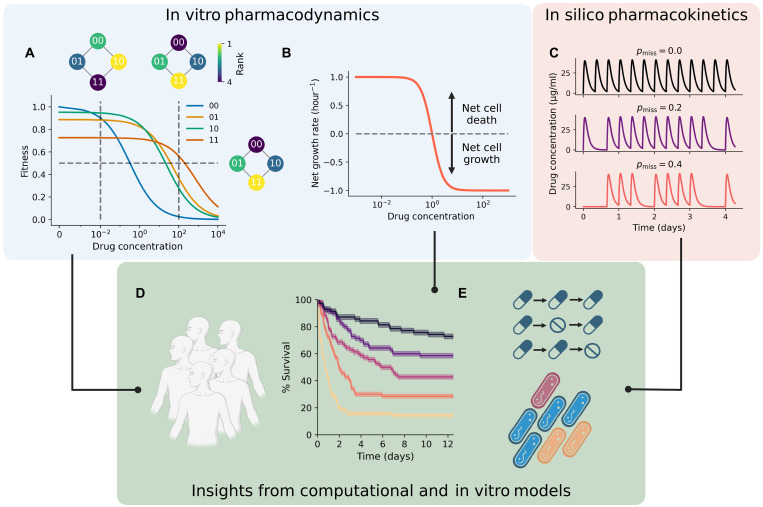
Fitness seascapes intrinsically model evolutionary trade-offs. (**A**) Fitness seascape parameterized with random synthetic data. The genotype with the highest IC_50_ (*11*) also exhibits the lowest drug-free growth rate, suggesting a trade-off, or cost, to resistance. Rank-order fitness landscapes based on the growth rate are shown inset, with the vertical dotted line at the corresponding drug concentrations. A fitness landscape based off of IC_50_ alone is also shown with a corresponding horizontal line. (**B**) Time-kill experiments are used to parameterize bacterial dose-response curves that include net cell death (net growth rate less than zero). (**C**) Patient dosing regimens are modeled with pharmacokinetic curves, which can be used to study patient nonadherence. The probability of a simulated patient missing a scheduled drug dose is given by $p_{\text{miss}}$ . (**D**) In this work, we simulate the pharmacokinetic characteristics of a wide variety of patients and study how infectious bacterial populations respond. Simulations are parameterized by integrated in vitro fitness seascapes and incorporate in silico pharmacokinetic curves. (**E**) We use in silico and in vitro models to evaluate the impact of drug dosing nonadherence on the probability of drug resistance.

Such trade-offs across environments are common in evolutionary medicine; drug resistance mechanisms often impose metabolic burdens or impair vital functions of the organism. When the drug selective pressure is removed, the sensitive genotype may have a higher relative fitness and the resistant genotype may go extinct (*20*, *21*). Moreover, disease agents in a patient will never experience a constant environment—the drug concentration will vary according to the pharmacokinetic profile, dosing schedule, and spatial distribution. Fitness seascapes extend the fitness landscape model by mapping both genotype and environment (i.e., drug concentration) to fitness (*22*–*28*). Here, we model fitness seascapes as collections of genotype-specific dose-response curves (Fig. 1A), similar to our previous work (*28*). This approach permits the modeling of fitness trade-offs across a range of drug concentrations, allowing us to model the evolution of resistance with realistic pharmacological considerations.

In this work, we describe an empirical fitness seascape in a clinically relevant *Escherichia coli* drug resistance model. We show that drug resistance in this model is associated with a substantial fitness trade-off, with a higher half-maximal inhibitory concentration (IC_50_) associated with a lower drug-free growth rate. We use this seascape and empirical dose-dependent death rates estimated from a time-kill assay to parameterize a computational model to explore intravenous (IV) and oral dosing regimens. In the IV antibiotic setting, we simulated patients treated for *E. coli* bacteremia, which carries a high rate of treatment failure with mortality estimates between 12 and 33% (*29*–*31*). In the outpatient setting, we simulated patients treated with oral antibiotics for less severe infections such as a urinary tract infection or pneumonia. We find that pathogen parameters such as mutation rate and population size affect the probability of treatment success in IV antibiotic regimens. We explore patient drug regimen nonadherence with oral dosing regimens and find that early drug regimen adherence is important for treatment success. We further validate this result by modeling treatment nonadherence in vitro, finding that missing the second dose results in treatment failure. Our work represents an application of an in vitro fitness seascape for studying clinical drug dosing regimens and serves as a bridge between evolutionary medicine and clinically relevant pharmacokinetic-pharmacodynamic models.

## RESULTS

### Fitness seascapes as a model for evolutionary trade-offs

Using synthetic data, we first explored how evolutionary trade-offs may be examined with fitness seascapes. Here, we define “trade-off” as a case where a genotype with a higher IC_50_ grows slower in the absence of drug than a genotype with a lower IC_50_. We also use “cost of resistance” interchangeably with trade-off. Figure 1A shows a synthetic fitness seascape that illustrates fitness trade-offs: Genotype 00 has the highest drug-free growth rate and exhibits the lowest IC_50_, while genotype 11 has the lowest drug-free growth rate and the highest IC_50_. This represents the case where drug resistance mechanisms impose some metabolic burden on the organism, such as the up-regulation of a protein or more energetically costly synthesis. Notably, trade-offs induce changes to the fitness landscape as the drug concentration changes, as shown in the inset rank-order fitness landscapes based on the growth rate. In contrast, the inset fitness landscape based on IC_50_ alone cannot intrinsically model trade-offs in this way.

### In vitro fitness seascape reveals evolutionary trade-offs in *E. coli*

Genetically engineered *E. coli* cell lines were provided by the Weinreich lab at Brown University (*10*). Details of the model system are provided in the Materials and Methods. Briefly, a combinatorially complete set of clinically relevant drug resistance point mutations was introduced into the *bla* gene encoding the ampicillin resistance (Amp_R_) β-lactamase protein. Sixteen genotypes were generated by engineering all possible combinations of four point mutations. We denote the presence or absence of a point mutation with a 0 (absence) or 1 (presence) in a binary string of length four, with each position in the string corresponding to a unique point mutation. 0000 denotes the wild-type *bla* gene; 0001 represents G238S, 0010 represents M182T, 0100 represents E104K, and 1000 represents A42G. Each of these point mutations is in the penicillin binding pocket of β-lactamase and serves to increase the rate of cefotaxime hydrolysis (*32*).

**Fig. 2.**
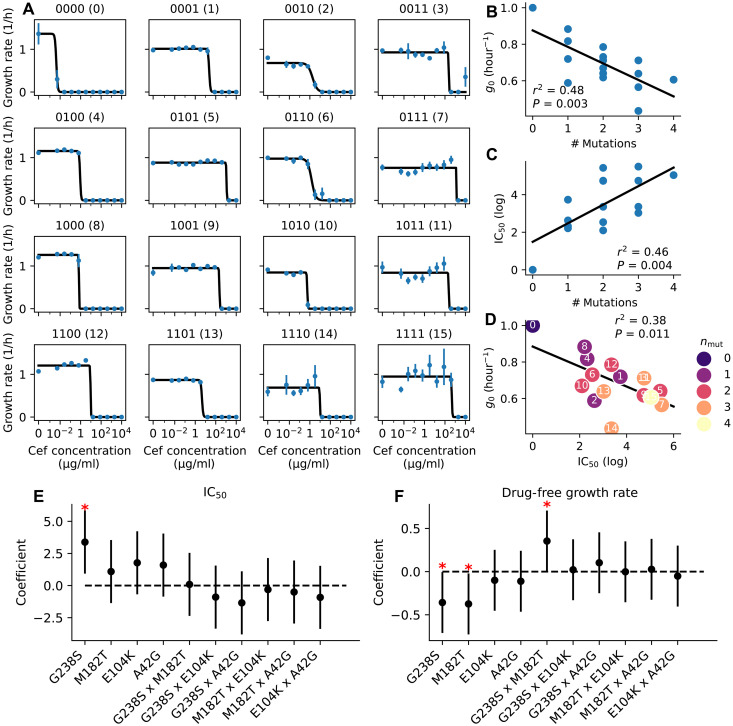
In vitro pharmacodynamics reveals fitness costs to resistance. (**A**) Cefotaxime (Cef) dose-response curves for all 16 genotypes. Growth rate estimates with SD are shown in blue ( N=8 replicates per condition). Pharmacodynamic curve fit is shown in black. h, hours. (**B**) Drug-free growth rate ( $g_{0}$ ) versus the number of mutations for all 16 genotypes. Linear regression is shown in black ( $r^{2}=0.48$ , P=0.003 ). (**C**) IC_50_ versus number of mutations. Linear regression is shown in black ( $r^{2}=0.46$ , P=0.004 ). (**D**) Drug-free growth rate versus IC_50_. Color of each data point indicates the number of mutations, while the number inset corresponds to the genotype. Linear regression is shown in black ( $r^{2}=0.38$ , P=0.011 ). (**E**) Multivariate regression assesing the impact of all four mutations and pairwise combinations on IC_50_. The red asterisk indicates a significant factor. Black bars indicate 95% confidence interval. (**F**) Multivariate regression assesing the impact of all four mutations and pairwise combinations on IC_50_. Red asterisks indicate significant factors. Black bars indicate 95%-ile interval.

We quantified dose-response curves for each genotype by estimating the growth rate as a function of drug concentration using an optical density (OD) assay (details in the Materials and Methods). The degree of drug resistance and drug-free growth rate varies widely across the 16 genotypes (Fig. 2). To understand the potential fitness costs to resistance-conferring mutations, we investigated the relationship between the number of mutations and the drug-free growth rate, finding that the two were inversely related (Fig. 2B; P=0.003 , $r^{2}=0.48$ ). We also found a positive correlation between the number of mutations and IC_50_ ( P=0.004 , $r^{2}=0.46$ ) and an inverse relationship between the drug-free growth rate and IC_50_ ( P=0.011 , $r^{2}=0.38$) (Fig. 2, C and D).

We next investigated whether particular resistance mutations were more costly than others, and, similarly, whether different resistance mutations conferred more resistance than others. To do this, we performed a multivariate linear regression with each resistance mutation as an independent predictor and either the drug-free growth rate or IC_50_ as the outcome. We also included all pairwise interaction terms in the analysis. Multivariate analysis revealed that G238S was the only mutation independently associated with IC_50_ (Fig. 2E). We found that both G238S and M182T were independently negatively associated with the drug-free growth rate, while the interaction term G238SxM182T was positively associated with the drug-free growth rate (Fig. 2F). These results suggest a reciprocal sign epistasis, where G238S and M182T are both deleterious on their own but advantageous to the drug-free growth rate when combined.

### Estimating dose-dependent death rates with a time-kill assay

We sought to apply our *E. coli* fitness seascape to computational studies of clinical drug dosing by modeling populations of bacterial cells treated with an antibiotic. To fully parametrize an agent-based algorithm, a robust model for cell death was necessary. However, experimental quantification of cell death rates was challenging. We found that we could not observe dynamics above the minimum inhibitory concentration (MIC) with our OD data—in the above-MIC range, we simply saw no cell growth. Previously published time-kill methods have several drawbacks, such as requiring hundreds of cell culture plates or suffering from residual drug carrying over into the cell count assay. To improve on the current status quo and better parameterize drug dose–dependent death rates, an accurate, facile, and low-footprint time-kill assay is needed.

**Fig. 3.**
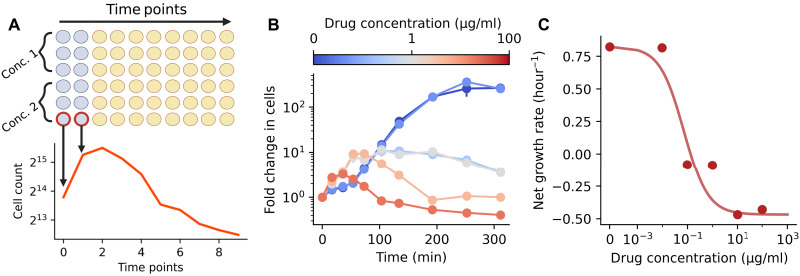
A time-kill assay for estimating dose-dependent death rates. (**A**) Illustration of the fluorescence-based time-kill assay used to parameterize bacterial death rates. Circles represent wells with bacterial culture in a 96-well plate; blue circles represent wells with alamarBlue (AB) added. Cell count over time for a single replicate is shown below in orange. (**B**) Cell count over time estimated from a time-kill assay for different concentrations of cefotaxime. SD bars may be smaller than the plotted data point. N=3 replicates for each time point. (**C**) Growth rate versus drug concentration estimated from data in (B). Solid line shows pharmacodynamic curve fit. SD bars are smaller than the plotted data point.

To address these shortcomings, we developed a fluorescence-based assay that uses resazurin as a cell viability indicator (details in the Materials and Methods). Resazurin is a dye that is blue and weakly fluorescent and is rapidly reduced to the pink and brightly fluorescent resofurin by viable cells. Briefly, a cell count to fluorescence calibration curve was generated and then used to estimate the cell count over time for cell cultures with different concentrations of cefotaxime. Ten time points were taken for each condition over the course of ~5 hours, with three technical replicates per condition. Summary cell count over time data is shown in Fig. 3A. We then estimated the maximum net change in cells, referred to as the “net growth rate,” for each drug concentration and fit these data to a pharmacodynamic curve (Fig. 3B). Last, we combined this pharmacodynamic relationship with the drug-free growth rate and IC_50_ estimated in Fig. 2A to obtain dose-response curves with positive and negative net growth rates.

### Pathogen mutation rate and population size affect the probability of treatment success

To understand what factors could contribute to treatment failure in *E. coli* infections, we applied our fitness seascape and time-kill data to computational models. To do this, we simulated patients undergoing a 10-day therapy regime with cefotaxime with an agent-based model: Fast Evolution on Arbitrary Seascapes (FEArS). FEArS models the number of dying, dividing, and mutating cells with a Poisson process (fig. S1). Mutations are modeled with an agent-based algorithm whereby mutating cells are randomly allocated to “adjacent” genotypes (genotypes that differ by Hamming distance 1). We parameterized FEArS with the fitness seascape and time-kill data described above to simulate infections with our *E. coli* model system. The agent growth rate in the sub-MIC regime (i.e., positive net growth rate regime) was parametrized with growth rate data from the fitness seascape, while the growth rate in the above-MIC regime was parameterized with time-kill data. We modeled patient serum pharmacokinetic profiles with a one-compartment model

$$c\left(t\right)=c_{\text{max}}\left(e^{-{\mathrm{tk}}_{\text{elim}}}-e^{-{\mathrm{tk}}_{\text{abs}}}\right)$$ (1)

where c(t) is the serum concentration over time, $c_{\text{max}}$ is the maximum achieved concentration, $k_{\text{elim}}$ is the drug clearance rate, and $k_{\text{abs}}$ is the drug absorption rate. N doses were modeled as a Dirac comb with the period T equivalent to the dosing frequency

$$\text{III}\left(t\right)=\sum_{d=0}^{N}\delta \left(t-\mathrm{dT}\right)$$ (2)

The final patient pharmacokinetic profile is then

$$c_{p}\left(t\right)=\text{III}\left(t\right)⊛c\left(t\right)$$ (3)

where ⊛ is the convolution operator.

**Fig. 4.**
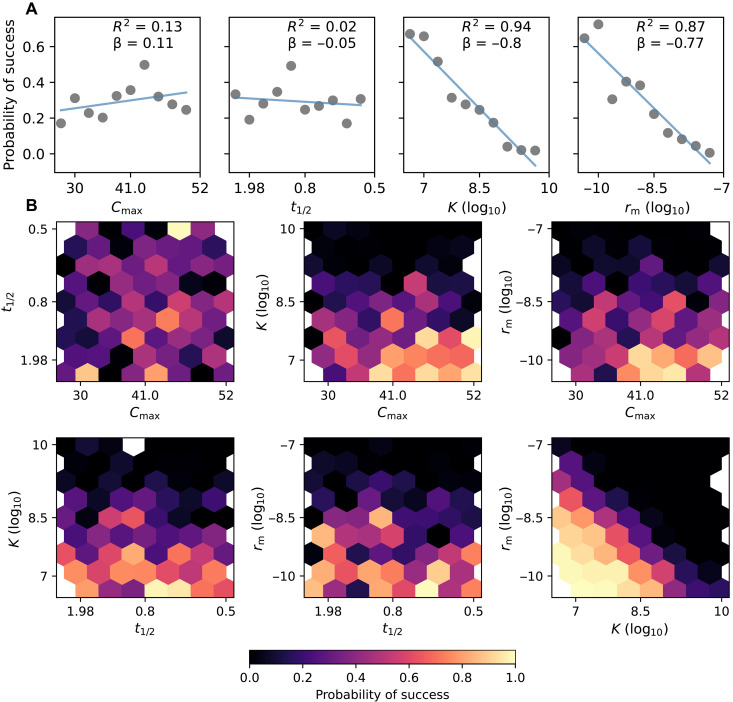
Impact of pharmacokinetics, carrying capacity, and mutation rate on treatment success. (**A**) Marginal distributions of probability of treatment success versus $t_{1/2}$ , $C_{\text{max}}$ , $r_{m}$ , and K for N=100 simulated patients per condition. Linear regression for each parameter is shown in blue. The coefficient of determination $R^{2}$ and coefficient of the linear regression β are shown inset. (**B**) Joint distributions of probability of treatment success. Treatment success was defined as 0 cells surviving by the end of the simulation. Dose frequency was one dose per 8 hours, duration of treatment was 10 days, and the simulations were allowed to reach equilibrium for 3 days before beginning the treatment. LHS was used to generate 300 sets of parameters.

Patient pharmacokinetics differ most notably in the maximum serum concentration $c_{\text{max}}$ and the drug half-life $t_{1/2}$ , which is governed by $k_{\text{elim}}$ in Eq. 1 (*33*). Infections can also differ in their mutation rate $r_{m}$ (*34*, *35*) and population size, here modeled as a carrying capacity K . To examine the interaction of all of these parameters, we used Latin hypercube sampling (LHS) to generate 300 sets of each of these parameters (*36*). LHS allows for uniform random sampling of a high-dimensional grid, ensuring that a wide and representative range of parameters and parameter combinations is tested. For each set of four parameters, we ran 100 simulations and computed the rate of treatment success (0 surviving cells by the end of the simulation). Parameter ranges tested are shown in Table 1. $c_{\text{max}}$ and $t_{1/2}$ had no discernible effect on treatment efficacy, while K and $r_{m}$ both strongly affected the treatment outcome (Fig. 4A). Multiple linear regression analysis revealed that there was a strong interaction between K and $r_{m}$ ( β=1.46 ); a summary of the linear regression coefficients is shown in table S3. We visualized this interaction by plotting the pairwise joint distributions between the parameters (Fig. 4B), finding that increasing K and $r_{m}$ jointly decreased the probability of success.

**Table 1.** Parameter ranges used for simulations with literature sources. N.A., not applicable.

Parameter	Description	Value range	Citation
$c_{\text{max}}$	Maximum serum concentration	30–52 μg/ml	(*33*)
K	Carrying capacity	10^7^–10^10^ cells	N.A.
$t_{1/2}$	Drug half-life	0.5–2 hours	(*33*)
$r_{m}$	Mutation rate	10^−10^–10^−7^ bp^−1^	(*34*, *35*)

We further examined the interaction between K and $r_{m}$ by plotting the rate of treatment success against average mutation supply, $K^{*}r_{m}$ , revealing a steep drop off in treatment success at one mutant per generation (fig. S2). Notably, prior work has found that high-mutation rate phenotypes are common among pathogenic *E. coli* (*37*), and the high mutation rate is strongly correlated with drug resistance in the clinic (*38*). Analyzing the evolutionary trajectory of the simulations revealed that almost all simulations in which there was treatment failure acquired the G238S mutation (allele 0), allowing for evolutionary escape (fig. S3). G238S is the mutation that confers the greatest change in IC_50_, on average, and has been found in many clinical samples. A high mutation supply produced a small population of genotypes 1 (0001) and 2 (0010) in equilibrium with genotype 0 before the onset of therapy, despite there being a fitness cost.

### Patient nonadherence contributes to treatment failure

Patient nonadherence is thought to contribute to treatment failure and antibiotic resistance in the ambulatory setting (*39*–*41*); studies have found that 30 to 45% of patients are nonadherent with their antibiotic regimen, with a large proportion of the nonadherence due to forgetfulness (*39*, *41*). To investigate this, we modeled varying drug elimination half-life $t_{1/2}$ , time to maximum drug concentration $t_{\text{max}}$ , and drug regimen nonadherence. We calculated the appropriate $k_{\text{elim}}$ and $k_{\text{abs}}$ from $t_{\text{max}}$ and $t_{1/2}$ and again applied Eq. 1 to model patient serum drug concentration.

**Fig. 5.**
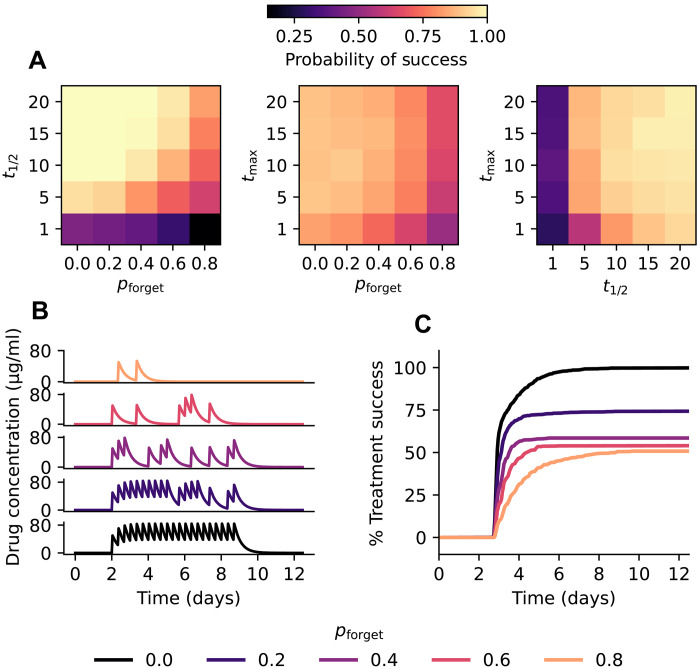
Patient nonadherence and drug half-life affect treatment success. (**A**) Joint distributions of probability of treatment success for $p_{\text{forget}}$ , $t_{1/2}$ , and $t_{\text{max}}$ . N=100 simulations per condition. (**B**) Example simulated patient pharmacokinetic curves for different values of $p_{\text{forget}}$ . (**C**) Time-to-event curves of treatment success for different rates of $p_{\text{forget}}$ . N=1000 simulations per $p_{\text{forget}}$ . The 95% confidence intervals are smaller than the widths of the plotted lines.

Patient nonadherence was modeled as a random process with each patient having a constant probability $p_{\text{forget}}$ of missing a scheduled dose. We modeled patients undergoing a 7-day treatment regimen using FEArS and measured the probability of treatment success for different combinations of the three parameters ( N=100 simulations per condition). There was a notable additive interaction between $t_{1/2}$ and $p_{\text{forget}}$ (Fig. 5A), with increasing $p_{\text{forget}}$ and decreasing $t_{1/2}$ jointly decreasing the rate of treatment success. In contrast, there was no clear relationship between $t_{\text{max}}$ and treatment success and no interaction between $p_{\text{forget}}$ and $t_{\text{max}}$ . To better understand what drives treatment failure in drug regimen nonadherence, we fixed $t_{1/2}$ and $t_{\text{max}}$ (6 hours and 1 hour, respectively) and simulated patients with varying rates of $p_{\text{forget}}$ ( N=1000 patients per condition). Representative drug concentration curves are shown in Fig. 5B. We found that different rates of $p_{\text{forget}}$ resulted in markedly different time-to-event curves, with higher rates of nonadherence leading to both lower rates of success and longer times to treatment success (Fig. 5C).

**Fig. 6.**
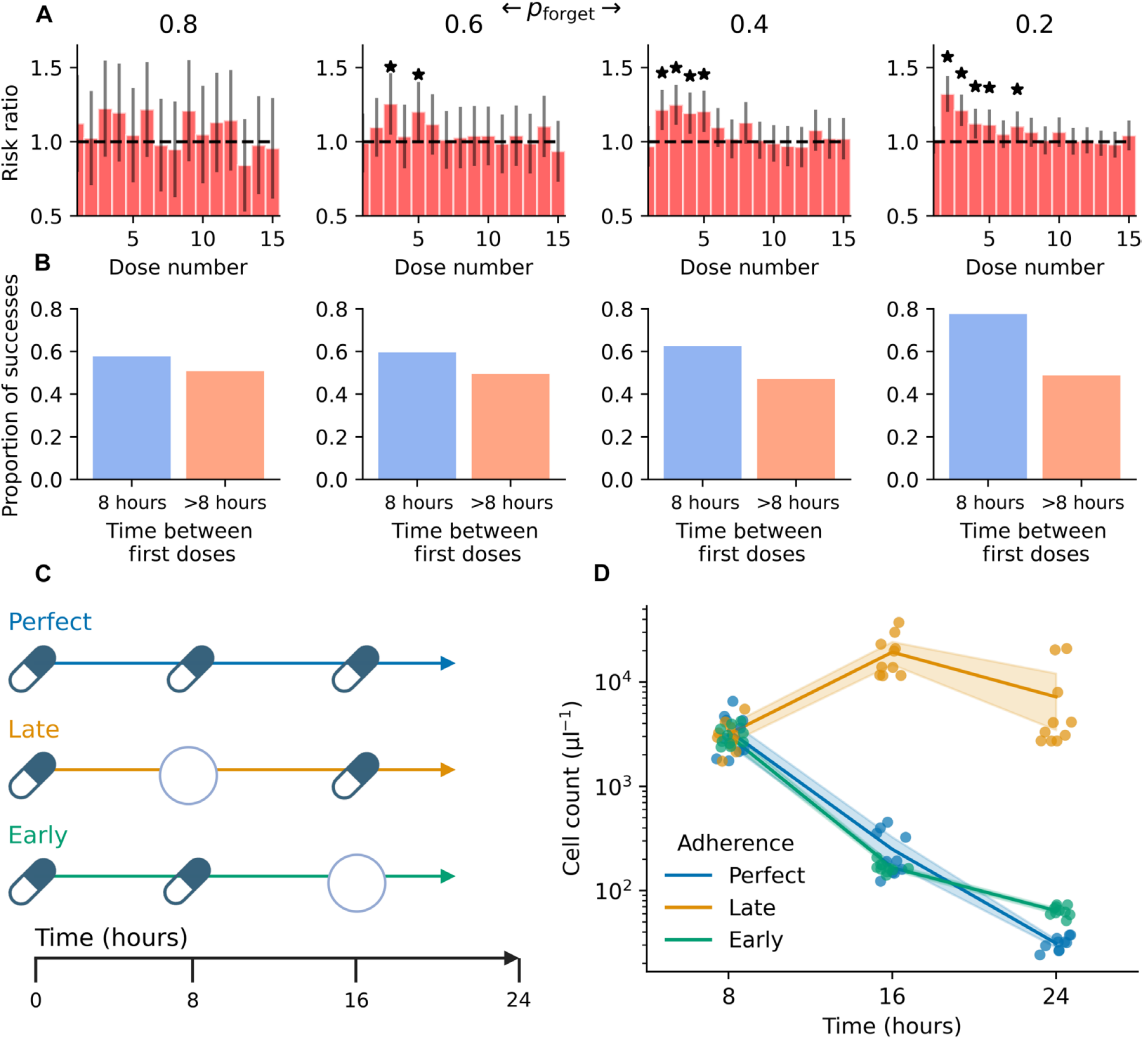
Early drug regimen adherence contributes to treatment success. Each column corresponds to a different $p_{\text{forget}}$ , labeled at the top. (**A**) Risk ratio for each dose based on the probability of treatment success. A higher risk ratio indicates a stronger association with treatment success. Error bars indicate 95% confidence intervals. Stars indicate where the 95% confidence intervals excludes 1. (**B**) Proportion of treatment success partitioned by regimens with 8 hours between the first two doses (blue column) and greater than 8 hours between the first two doses (orange column). N=1000 simulations per $p_{\text{forget}}$ . (**C**) Illustration of in vitro drug dosing experiment. Perfect refers to all three correctly administered drug doses; late refers to a missed second dose and correctly administered third dose (i.e., late adherence); early refers to a correctly administered second dose and missed third dose (i.e., early adherence). (**D**) Cell count over time estimated with AB for three conditions in (C). Shaded region shows 95% confidence intervals for each time point. N=8 replicates per condition.

We hypothesized that there may be patterns in patient nonadherence that affect treatment success. We calculated the risk ratio of each individual drug dose and found that, for moderate rates of nonadherence ( $p_{\text{forget}}\leq 0.6$ ), early drug doses were particularly important for treatment success (Fig. 6A). In this case, a higher risk ratio indicates that a particular dose was more important for treatment success. On the basis of this finding, we calculated the probability of treatment success partitioned by time between the first two doses, either equal to 8 hours or greater than 8 hours between the first two doses (Fig. 6B). We found that regimens where the second dose was administered exactly 8 hours after the first had a higher rate of success compared to regimens where the second dose came greater than 8 hours after the first, and this difference increased as the nonadherence rate decreased. This suggests that at low rates of nonadherence, appropriately timing the first two doses is critical for treatment success. We also tested whether the total number of doses correctly administered corresponded to treatment success for a given $p_{\text{forget}}$ , finding that the average difference in total doses between successful and unsuccessful regimens was less than 1 (fig. S4). This observation suggests that the total number of doses is not a meaningful independent predictor of treatment success.

### In vitro validation supports importance of early adherence

To further explore the computational finding that early nonadherence affects treatment success, we modeled nonadherence in vitro and studied the impact of different dosing regimens. Using cocultures of genotypes 0, 1, and 2 to generate standing genetic heterogeneity, we dosed cultures in 96-well plates with three different cefotaxime regimens (Fig. 6C): perfect, representing 100% adherence (three successive doses with 8-hour intervals); late, representing late adherence (i.e., missing the second dose); and early, representing early adherence (i.e., missing the third dose). Cultures were passaged every 8 hours, and the drug dose was adjusted during passaging according to the dosing regimen. Note that the cells were not resuspended in fresh media during passaging, so there was some drug carry-over; this results in a difference (~1 μg/ml) in drug concentration between the perfect adherence regimen and the nonadherence regimens and likely does not affect the qualitative interpretation of the experiment. After each 8-hour interval and before passaging, plates were scanned with alamarBlue (AB) to estimate the cell count over time. In the perfect adherence condition, the cell count dropped continuously after each successive dose, demonstrating robust treatment response (Fig. 6D). In the early adherence condition, the cell count mirrored that of the perfect adherence condition despite missing the third dose at 16 hours, suggesting that the two early doses strongly reduced the viable cell count. In contrast, in the late adherence condition, there was continued cell growth after the third scheduled dose—after missing the second dose, the population rebounded to ~10^4^ cells/μl. Following the third administered dose at 16 hours, the population count did not fall appreciably, demonstrating drug resistance. These results support the finding that two successive doses at the beginning of the regimen are vital to prevent drug resistance and successfully inhibit population growth.

## DISCUSSION

Here, we have described an in vitro fitness seascape of a clinically relevant drug resistance model and used it to generate insights into clinical drug dosing regimens. By quantifying the growth rate of genotypes representing a combinatorially complete set of drug resistance mutations, we have found that resistance-conferring mutations in the TEM β-lactamase gene impose substantial fitness costs in *E. coli*. This finding aligns closely with a meta-analysis of fitness costs to drug resistance in bacteria, where the magnitude of the fitness cost was found to be positively correlated with the MIC, which is an indicator of drug resistance (*42*). Allele-level analysis revealed that G238S and M182T are both negatively correlated with the drug-free growth rate, but the interaction term G238SxM182T was positively correlated, suggesting reciprocal sign epistasis. This interaction may relate to the biochemical function of the M182T mutation, which stabilizes other drug resistance mutations in TEM including G238S. This finding may also help explain why G238S and M182T are often found together in clinical TEM isolates (*43*). We also found that G238S is significantly associated with IC_50_—previous biochemical and computational studies of the TEM β-lactamase have shown that the G238S mutation is associated with the highest increase in cefotaxime hydrolysis and is likely a major driver of clinically relevant drug resistance (*44*). Together, these genotype- and allele-level findings provide insights into the cost of drug resistance in bacteria.

We used this seascape model to explore intravenous and oral drug dosing regimens with computational agent-based modeling. We found that parameters that contribute to mutation supply—mutation rate and population size—most strongly influence the probability of treatment success in our model of *E. coli* bacteremia. In the simulated outpatient setting with oral antibiotic dosing, we found that drug regimen nonadherence is associated with treatment failure, but early drug regimen adherence contributes to treatment success. We further validated this finding in vitro by studying the impact of the time between drug doses, finding that missing the second dose in a series (i.e., “late” adherence) strongly correlates with evolutionary escape.

Mathematical and computational modeling of within-host evolutionary dynamics has a long and rich history, especially in applications to long-term infections such as HIV and tuberculosis (*45*–*48*). Here, we have expanded on this prior work in several key ways. First, we parameterized our simulations with an empirical fitness seascape, enabling us to study evolutionary dynamics with a clinically relevant drug resistance model. By quantifying growth rates, and thus inherently capturing fitness trade-offs, fitness seascapes provide a more realistic model for selection under varying drug concentrations. In addition, we developed a time-kill assay to estimate dose-dependent bacterial death rates, allowing us to more accurately simulate cell death in our agent-based models. Combining empirical genotype-specific growth rates and drug dose–dependent death rates reduces the number of free biological parameters in our model, increasing the external validity of our findings. Furthermore, we used a hybrid agent-based algorithm (FEArs) for stochastic simulations. This choice enabled efficient computational experiments, allowing for a large number of parameter combinations and replicates while maintaining important biological constraints, even for very small population sizes. This innovation allows us to examine population extinction and evolutionary rescue from extinction, which is important for modeling the emergence of drug resistance in the clinic. In addition to the methodological advances, our work provides insights into the somewhat underappreciated evolutionary dynamics of short-term infections in the clinic. Our findings suggest that the emergence of de novo drug resistance mutations during the course of therapy may contribute to treatment failure in both the inpatient and outpatient settings with timescales of days to weeks.

A natural extension of this work is in modeling antibiotic cycling to avoid resistance in bacteria. Much interest has been given to optimizing antibiotic cycling in both computational studies with fitness landscapes (*9*, *11*, *49*–*52*), in vitro studies (*53*), and clinical trials (*54*). In these models, different antibiotics are associated with different fitness landscapes. Each landscape is, in essence, a tool that can be used to push an evolving population in a desirable direction. By conceptualizing these landscapes as seascapes, we can simultaneously increase the accuracy of our models and the number of distinct fitness landscape topographies available to use for the purposes of steering evolution (*6*, *51*). Each individual drug may produce several different changes in genotype fitness rank as a function of antibiotic concentration.

Optimizing drug dosing regimens is another natural application of our work. By varying the timing and dose of an antibiotic therapy, we may discover parameter ranges that reliably avoid resistance in silico. Then, using empirical knowledge of patient preferences (*55*, *56*), we may deliver dosing regimens that are both amenable to patient adherence and successful elimination of the infection.

There are several important limitations to this work. The four-allele fitness seascape quantified here represents only a small set of possible resistance mutations. For instance, off-target mutations in genes regulating drug efflux pumps or cell metabolism may also contribute to resistance. A more robust method for generating genotype-fitness mappings in an unbiased way will likely be necessary for generalizing these results. Furthermore, the evolutionary model used here only represents well-mixed *E. coli* growing in vitro—we do not model immune system interactions, spatial considerations, or frequency-dependent fitness effects. Last, we did not explore differential drug penetration into different tissues, which has been found to promote resistance (*48*, *57*).

Apart from the specific findings of our work, the fitness seascape model and concept of fitness trade-offs more generally have larger implications for molecular evolution in varying environments (*26*, *58*, *59*). The fitness seascape model suggests that selection changes as a function of environmental variables—considering that environmental variables can change over time, selection itself can then change over time. While this is not necessarily an unexpected conclusion, this phenomenon calls into question the utility of fitness as a scalar, fixed variable. The canonical fitness landscape model in infectious disease posits that higher fitness proteins are those that confer a greater degree of drug resistance and that evolution will trend toward these higher fitness proteins. In contrast, the fitness seascape model indicates that evolution may not proceed in such a straightforward manner, and selection may be difficult or impossible to predict based on measures of drug resistance alone (*58*). Furthermore, the shape of dose-response curves may change the standing genetic heterogeneity in a population, thus influencing the progression of molecular evolution. For instance, a highly costly mutation that confers a great deal of drug resistance may be exceedingly unlikely to emerge due to selection against the mutation at low drug concentrations. Others have shown that patterns such as global epistasis are also shaped by complex interactions of dose-response curves (*60*).

Our work represents an application of an empirical fitness seascape to predicting the emergence of resistance in the clinic. Our results suggest that explicit modeling of evolutionary trade-offs is important for predicting evolution under time-varying drug concentration and that this modeling framework may be useful for optimizing drug dosing regimens in silico and designing adaptive therapies.

## MATERIALS AND METHODS

### Model system

Genetically engineered *E. coli* DH5α strains were provided by the Weinreich Lab at Brown University. Details of the genetic engineering are provided by Weinreich *et al.* (*10*). A description of each of the 16 genotypes and their clinical designation, if any, are shown in table S1. We searched National Institutes of Health (NIH) Basic Local Alignment Search Tool (BLAST) for each β-lactamase sequence to see whether it had been previously identified in clinical contexts, noting its clinical designation if applicable.

Cells were cultured from frozen glycerol stock in LB broth. Overnight cultures were grown with tetracycline (10 μg/ml; MP Biomedicals). Cefotaxime (Thermo Fisher Scientific) was prepared according to manufacturer’s specifications and stored at −20°C in deionized water.

### Growth rate estimation

We generated an OD to cell count calibration curve using a colony-forming unit (CFU) assay. *E. coli* cells were inoculated from frozen glycerol stock in 3-ml LB broth and incubated overnight at 37°C and 220 rpm. A series of twofold dilutions with fresh media was made in a 96-well plate, and the OD was measured. Samples corresponding to 1-, 4-, 16-, and 64-fold dilutions were then used to quantify the cell count with a CFU assay. Samples were diluted 100,000-fold, and 50 μl was deposited on agar plates in triplicate. After overnight culture, individual colonies were counted by eye. We then generated a calibration curve by fitting a linear regression to the log-transformed OD and cell count data.

To measure the fitness seascape, strains were inoculated into LB broth + tetracycline (10 μg/ml) and grown up overnight in a bacterial shaking incubator set to 37°C and 225 rpm. Sixteen plates, one plate per genotype, were prepared with a fivefold cefotaxime dilution scheme and one row free of antibiotic. The plates were organized such that there were eight experimental replicates per drug concentration. One microliter of bacteria was inoculated into each well, after which plates were sealed with adhesive film. Plates were then incubated at 37°C with no shaking and transferred by hand to a Tecan Spark microplate reader for OD_600_ readings every 2 hours for 20 hours. Plates were shaken at 510 rpm for 10 s before OD_600_ readings.

After background subtraction, the time series OD data were converted to the cell count using the calibration curve. We then estimated the growth rate for each condition using an approach adapted from Mira *et al.* (*61*). For each growth curve, the data were log-transformed, the slope of each measurement interval was computed, and the interval containing the maximum rate of change was identified. Intervals surrounding the maximum rate of change were then included for further analysis if their slope was within 0.7× the maximum slope. We then used these identified intervals to compute a linear regression to estimate the growth rate.

To estimate the pharmacodynamic relationship, we fit the following dose-response equation to the estimated growth rates

$$g\left([c]\right)=\frac{g_{\text{drugless}}}{1+e^{\left({\text{IC}}_{50}-\text{log}\left([c]\right)\right)/\nu }}$$ (4)

where [c] is the drug concentration, $g_{\text{drugless}}$ is the growth rate in the absence of drug, ${\text{IC}}_{50}$ is the drug concentration that inhibits the growth rate by 50%, and ν is the Hill coefficient that determines the steepness of the curve. All data analysis was done in Python using the SciPy package (*62*).

### Genotype- and allele-level analysis

To investigate the relationship between the IC_50_ and drug-free growth rate on the scale of individual genotypes, we performed linear regressions on the IC_50_ and drug-free growth rate as functions of the total number of drug resistance mutations. Then, we performed a linear regression on the drug-free growth rate as a function of IC_50_. To investigate, allele-level effects, we set up a linear regression with each allele and each pairwise interaction term as predictors and either IC_50_ or drug-free growth rate as the outcome. The presence or absence of an allele was coded as a 1 or 0, respectively. Univariate statistical analyses were performed using the SciPy Python package (*62*). Multivariate regressions were performed using the statsmodels Python package (*63*). All data and analysis code can be found at https://doi.org/10.5281/zenodo.14969809.

### Time-kill assay

We developed a time-kill assay to estimate drug dose–dependent death rates using AB cell viability reagent (Thermo Fisher Scientific).

#### Generating calibration curves

We generated a curve to estimate the bacterial cell count from AB fluorescence using a two-step procedure. First, we generated a cell count to OD calibration curve. *E. coli* cells were inoculated from frozen glycerol stock in 3-ml LB broth and incubated overnight at 37°C and 220 rpm. We made a series of twofold dilutions of the culture in fresh media using 90 μl of cell culture per well in a clear, polystyrene 96-well plate, with rows B to G serving as technical replicates and columns 2 to 10 comprising the dilution gradient. Column 11 was used for background estimation by adding fresh medium only. OD was measured using a microplate reader, and samples from row B, columns 2, 4, 6, and 8 were subjected to a CFU assay to quantify cell count. These samples corresponded to 1-, 4-, 16-, and 64-fold dilutions from the initial culture. We diluted each sample by 100,000-fold and plated 50 μl onto agar plates in triplicate. Colonies were manually counted after overnight incubation at 37°C. We then background-subtracted the OD data and fit a linear model to the log-transformed OD and cell count data. We used the slope and *y* intercept to estimate the cell count from OD using a linear fit of the log-transformed cell count and OD_600_ values.

To estimate fluorescence, 10 μl of AB was added to each well of the same 96-well plate (excluding row B used for the CFU assay), and the plate was incubated for 30 min at 37°C with 220 rpm shaking. After incubation, fluorescence was measured with a microplate scanner (excitation filter, 540 nm; emission filter, 590 nm). After background subtracting, we used the OD-CFU calibration curve above to estimate the cell count as a function of relative fluorescence units (RFU_30_). As before, we estimated the calibration curve with a linear regression on the log-transformed data.

#### Estimation of dose-response curves with time-kill assay

Three 96-well plates were prepared with each plate containing two conditions and three experimental replicates per condition. Rows B to G were used for experimental replicates, while columns 2 to 11 were used for different time points. To optimize the time each condition spent in the dynamic range of the assay, we used different starting concentrations of cells for the two above- and below-MIC conditions. For the above-MIC conditions (conditions where the cell count was not expected to increase), we initialized the experiment with ~2 × 10^5^ cells/ml, which we obtained by diluting the overnight culture by 4× with fresh media. For the below-MIC conditions, we initialized the experiment with ~2 × 10^3^ cells/ml by diluting the overnight culture by 100×. All experiments were initialized after 1 hour of preincubation after diluting the overnight culture. We prepared a stock solution of cefotaxime (20,000 μg/ml) following the manufacturer’s instructions. To ensure that each condition was exposed to drug at roughly the same time, 10 μl of the cefotaxime solution (Thermo Fisher Scientific) was added to the wells for each condition first, followed by 90 μl of cell culture. The wells on the outer boundary of the plate were left empty, as fluorescence readings were found to be unreliable for these wells.

At time 0, 10 μl of AB was added to column 2 of each plate, and plates were incubated for 30 min at 37°C with 220 rpm shaking. After 30 min, fluorescence was measured with the microplate scanner. Ten microliters of AB was added to column 3 15 min after adding AB to column 2, and plates were incubated for 30 min before performing the corresponding fluorescence scan. This process was repeated for columns 4 to 11, with plates being scanned 30 min after adding AB to each column. We used 15-min time intervals between adding the AB in columns 2 to 6, 30-min time intervals for columns 6 to 8, and 1-hour time intervals for columns 8 to 11. This approach resulted in sampling time points of roughly 0, 15, 30, 45, 60, 90, 120, 180, 240, and 300 min. This sampling scheme allowed us to observe the short- and long-term dynamics of the change in the cell count over time. We applied the RFU_30_–cell count calibration curve from above to estimate the cell count at each time point.

#### Net growth rate estimation

Estimating the parameter of interest from time-kill data is a challenge, especially during cell death. While others have used parametric equations constructed from first principles (*64*), we found overfitting to be an impassable challenge. Furthermore, we observed a variable death rate in our high drug concentration conditions. Hence, a simple model with fewer parameters may be a more robust approach. As an illustration, we sought to estimate the maximum rate of change in cells using a linear regression on the log-transformed cell count data.

To choose a subset of the data that balanced linearity with including as much data as possible, we defined the following objective function

$$l\left(i,j\right)=\left\{\begin{array}{cc} \infty , & \text{if}\;R_{i,j}\notin B\\ 1-R_{i,j}^{2}+\lambda /n^{2}, & \text{otherwise} \end{array}\right.$$ (5)

where i and j are the start and end indices for the subset, $R_{i,j}$ is the correlation coefficient for the subset, $R_{i,j}^{2}$ is the coefficient of determination, n is the number of points included in the subset, λ is a regularization parameter, and B is the boundary-defining set. Since $1-R_{i,j}^{2}$ is strictly between 0 and 1, we set λ equal to 1. We also defined a boundary set B for the estimated slope of the regression based on whether the cell count was increasing or decreasing. For instance, for the drug-free condition, the slope must be greater than 0 since the cell count increased, so B={x:x>0} . If $R_{i,j}$ is not in the set B , then the loss function is set to infinity. We then minimized l(i,j) for each dataset by an exhaustive search of all subsets larger than two elements. For the optimization step, time and the cell count for each dataset were normalized between 0 and 1. We then used the subset identified with this process to estimate the maximum rate of change in the cell count using a linear regression.

All regressions were performed with the SciPy Python package. All code and data required for reproduction are available in the repository: https://doi.org/10.5281/zenodo.14969809.

### Integrated fitness seascape parametrization

We parameterized genotype-specific dose-response curves with the following pharmacodynamic equation

$$g\left([c]\right)=g_{\text{max}}+\frac{\left(g_{\text{min}}-g_{\text{max}}\right)*{\left[c\right]}^{\nu }}{{\text{IC}}_{50}^{\nu }+{\left[c\right]}^{\nu }}$$ (6)

where $g_{\text{max}}$ is the maximum estimated growth rate, $g_{\text{min}}$ is the minimum growth rate (i.e., maximum net loss rate), and ν is the Hill coefficient. We used time-kill data from genotype 0 to estimate $g_{\text{min}}$ and ν and applied this to all of the dose-response curves. We used the drug-free growth rate and ${\text{IC}}_{50}$ estimated from Fig. 2 to generate genotype-specific dose-response curves. These dose-response curves were then used to parametrize FEArS for computational modeling.

### Pharmacokinetic nonadherence modeling

We modeled patient nonadherence by convolving an impulse train representing correctly administered doses with the two-compartment pharmacokinetic model (Eq. 1). Formally, this is done by generating a set ( D ) of numbers representing the time of each scheduled drug dose. Then, for each patient, a random subset of drug doses is removed from D , with each drug dose having a constant probability $p_{\text{forget}}$ of being removed, resulting in the set of drug doses that the simulated patient correctly self-administered ( $D_{a}$ ). A Dirac comb is generated using $D_{a}$ with the period T equal to the time between drug doses

$${\text{III}}_{a}\left(t\right)=\sum_{d\in D_{a}}\delta \left(t-\mathrm{dT}\right)$$ (7)

and the resulting impulse train is convolved with the pharmacokinetic model in Eq. 1 to generate the simulated patient’s serum drug concentration

$$c_{\text{patient}}\left(t\right)=c\left(t\right)⊛{\text{III}}_{a}\left(t\right)$$ (8)

where ⊛ is the convolution operator.

### In vitro drug regimen nonadherence model

We developed an in vitro model to better understand the impact of early versus late nonadherence. Rows B to D in a 96-well plate were used as experimental conditions, with columns 2 to 11 being technical replicates. *E. coli* genotypes 0, 1, and 2 were cultured overnight as described previously. Ten microliters of genotype 0 culture was added to 90 μl of fresh media in each experimental well for a 1:10 dilution. Cultures of genotypes 1 and 2 were diluted 1:10 in fresh media before adding 1 μl to each well for a 1:103 dilution. Then, wells were dosed with either cefotaxime (10 μg/ml) or media control. Every 8 hours, the cells were passaged to a new 96-well plate in a 1:10 dilution, and the drug concentration was updated according to the schedule in table S2. Cells were not resuspended in fresh media before passaging, so some residual drug was carried over into the subsequent wells. However, this small amount of residual drug represents a difference of at most 1 μg/ml between conditions and thus does not affect the qualitative result of the experiment. After passaging, 10 μl of AB was added to each well, and the cell count was estimated using the method described above. An additional 24-hour AB scan was completed after the 16-hour passage.
