# Supplementary material for: Fitness seascapes are necessary for realistic modeling of the evolutionary response to drug therapy
Source: Sci Adv. 2025 Jun 11;11(24):eadv1268. doi: 10.1126/sciadv.adv1268 (PMC12153978; doi:10.1126/sciadv.adv1268)
Supplement: Supplementary file 1 — Supplementary Text Figs. S1 to S4 Tables S1 to S3 [file sciadv.adv1268_sm.pdf]

Science Advances
AAAS

Supplementary Materials for

Fitness seascapes are necessary for realistic modeling of the evolutionary response to drug therapy

Eshan S. King *et al.*

Corresponding author: Eshan S. King, esk81@case.edu; Jacob G. Scott, scottj10@ccf.org

Sci. Adv. **11**, eadv1268 (2025)
DOI: 10.1126/sciadv.adv1268

This PDF file includes:

Supplementary Text
Figs. S1 to S4
Tables S1 to S3

Supplementary Text

FEArS: an agent-based modeling platform

We developed FEArS, Fast Evolution on Arbitrary Seascapes, to simulate bacterial populations responding to drug with our fitness seascape and time-kill data. An overview of the core evolutionary algorithm is shown in **Fig. S1**.

Mutation supply impacts probability of treatment success

We performed a Monte Carlo simulation using Latin Hypercube Sampling to investigate the impact of maximum serum drug concentration c_{max}, drug half life $t_{1/2}$, carrying capacity K, and mutation rate r_m on the probability of treatment success in the setting of inpatient *E. coli* bacteremia treated with IV antibiotics. We used a multiple linear regression to analyze the relative importance of each of the parameters on the outcome, shown in **Table S3**. We found that K and r_m were both independently and jointly associated with treatment success.

We investigated the impact of the average mutation supply, Kr_m, on the probability of treatment success (**Fig S2**). Above ~1 mutant per hour, there was a very low probability of treatment success. We further investigated the genotype-specific cell count for different mutation supply scenarios (**Fig S3**), finding that for higher rates of mutation supply, drug resistant genotypes exist at a low population count in equilibrium with the wild-type.

Total administered doses is not a meaningful predictor of treatment success

We sought to determine whether the total number of doses correctly administered may correspond to treatment success (**Fig. S4**). While there are slight differences between the total doses between regimens that resulted in failure and success, the difference in the mean is less than one dose on average, and thus not likely to be clinically significant.

Table S1: **Engineered *E. coli* genotypes and their clinical designations.** Columns A42G, E104K, M182T, and G238S each correspond to the four β-lactamase point mutations in the model system. A 1 or 0 in the column for a mutation indicates the presence or absence of that mutation in the genotype.

**Genotype #**	**Clinical designation**	**A42G**	**E104K**	**M182T**	**G238S**
0	TEM-1	0	0	0	0
1	TEM-19	0	0	0	1
2	TEM-135	0	0	1	0
3	TEM-20	0	0	1	1
4	TEM-17	0	1	0	0
5	TEM-15	0	1	0	1
6	TEM-106	0	1	1	0
7	TEM-52	0	1	1	1
8	None	1	0	0	0
9	None	1	0	0	1
10	None	1	0	1	0
11	None	1	0	1	1
12	None	1	1	0	0
13	None	1	1	0	1
14	None	1	1	1	0
15	None	1	1	1	1

Table S2: **Drug dosing schedule.**

Timepoints	**0 hr**	**8 hr**	**16 hr**
**Row B**	10 μg/mL	10 μg/mL	10 μg/mL
**Row C**	10 μg/mL	0	10 μg/mL
**Row D**	10 μg/mL	10 μg/mL	0

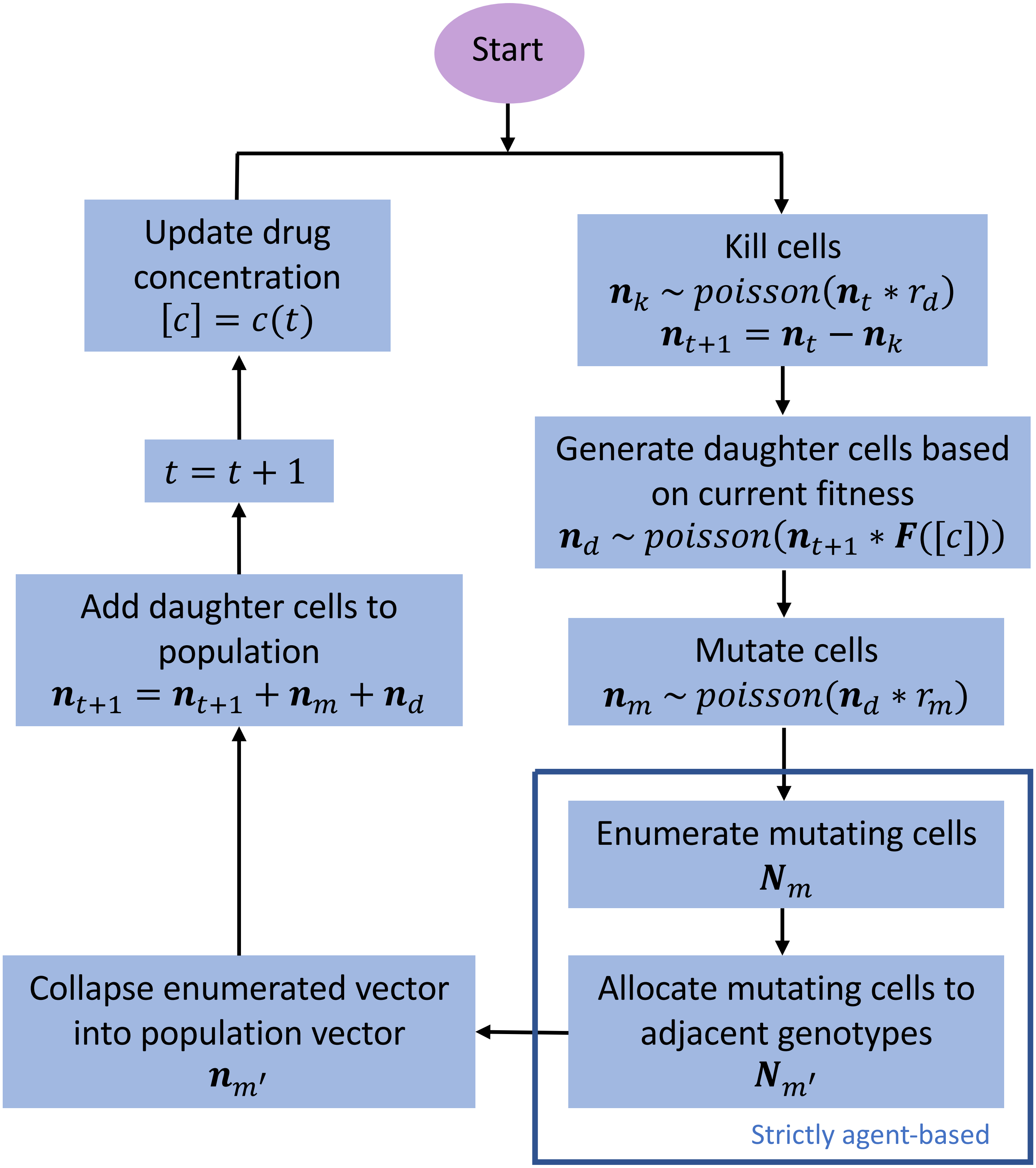

Figure S1: **FEArS evolution simulation flowchart.**

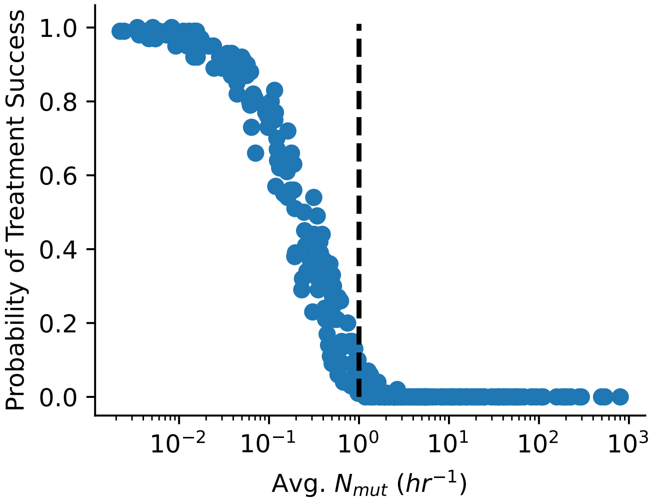

Figure S2: **Mutation supply versus probability of treatment success.** Mutation supply (Avg N_{mut}) is the product of the mutation rate and the carrying capacity. Vertical line indicates 1 mutant per hour.

Table S3: **Multiple linear regression analysis and interactions for simulated bacteremia treated with IV antibiotics.**

Variable	Regression Coefficient (β)	Standard Error
Intercept	1.6632	0.070
c_{max}	-0.0124	0.091
$t_{1/2}$	-0.3797	0.086
K	-1.6488	0.087
r_m	-1.6351	0.087
$c_{max} \times t_{1/2}$	0.0864	0.091
$c_{max} \times K$	-0.0276	0.092
$c_{max} \times r_m$	-0.0680	0.095
$t_{1/2} \times K$	0.2147	0.088
$t_{1/2} \times r_m$	0.2399	0.090
$K \times r_m$	1.4565	0.091

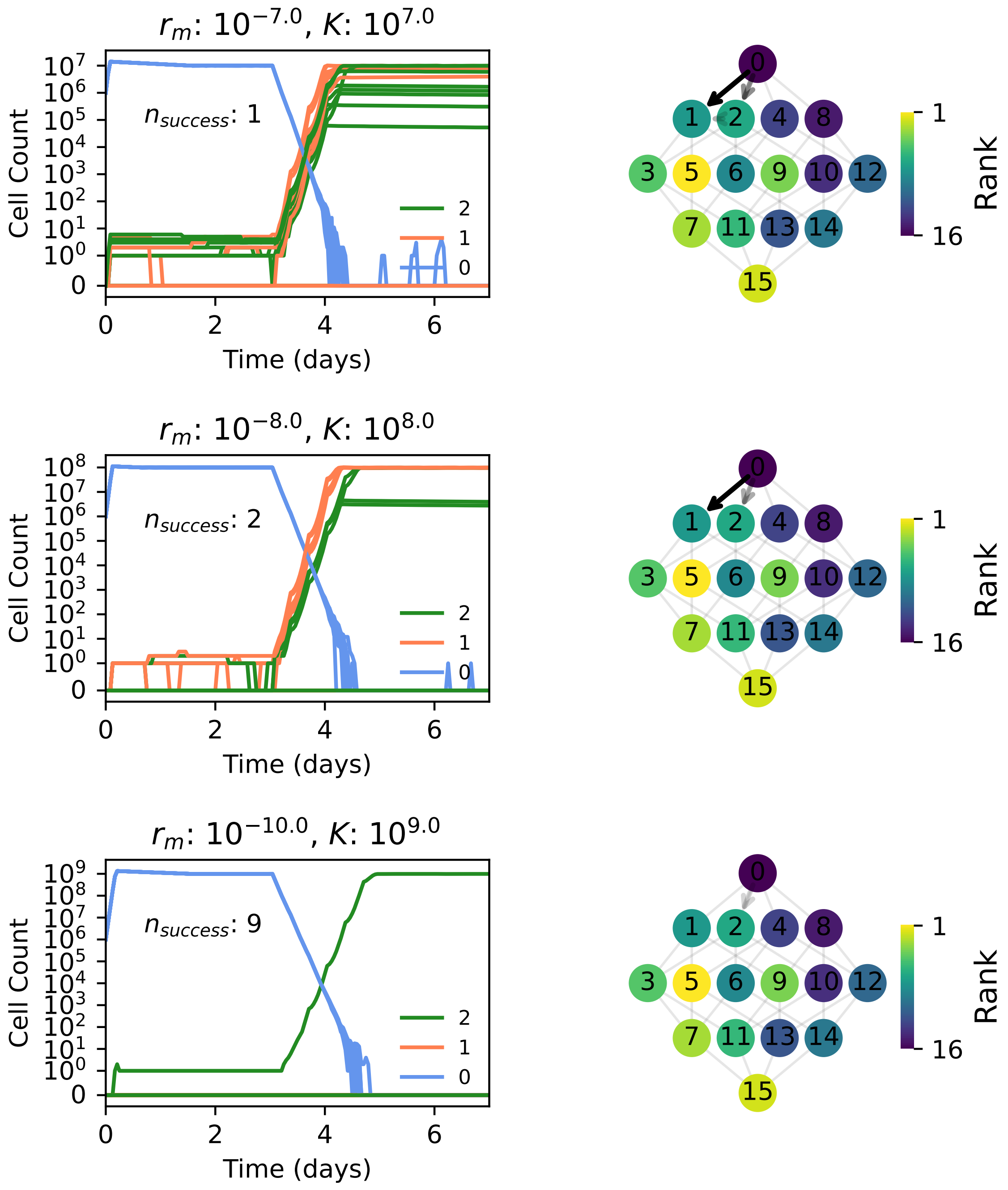

Figure S3: **Higher mutation supply increases probability of resistance.** Left column: cell count over time for genotypes 0, 1, and 2 for three combinations of r_m and K. Top two rows correspond to mutation supply ˜1 per cell division; bottom row corresponds to mutation supply ˜0.1 per cell division. Right column: evolutionary trajectories corresponding to the data on the left. Arrow opacity corresponds to the number of simulations following a given trajectory, with a maximum of six trajectories for the darkest lines and one trajectory for the lightest lines. $N = 10$ simulations per parameter set; treatment began at $t = 3$ days.

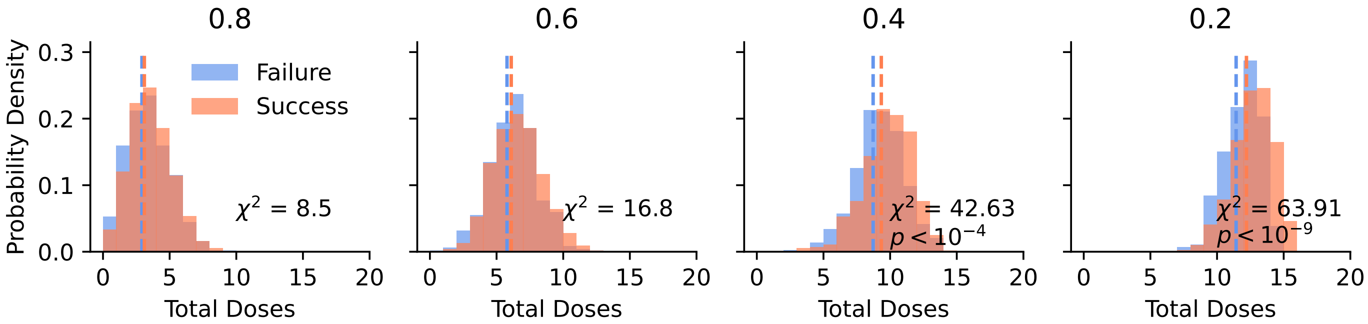

Figure S4: **Total number of administered doses is associated with treatment success.** Columns correspond to different rates of p_{forget}, labeled at the top. Dotted vertical lines indicate the mean of the distributions. χ-squared statistic and p-values are shown inset. N = 1000 simulations per p_{forget}.
